# A Set of Rapid Diagnostic Tool for *Babesia microti* Infection

**DOI:** 10.1002/jcla.70102

**Published:** 2025-09-19

**Authors:** Yanan Bai, Shangdi Zhang, Qindong Liang, Xinxin Zhang, Zeen Liu, Yuxin Ye, Jianxun Luo, Hong Yin, Chongge You, Guiquan Guan, Jinming Wang

**Affiliations:** ^1^ Laboratory Medicine Center The Second Hospital & Clinical Medical School, Lanzhou University Lanzhou Gansu P. R. China; ^2^ State Key Laboratory for Animal Disease Control and Prevention, College of Veterinary Medicine, Lanzhou University, Lanzhou Veterinary Research Institute, Chinese Academy of Agricultural Sciences Lanzhou Gansu China; ^3^ Key Laboratory of Veterinary Parasitology of Gansu Province, Gansu Province Research Center for Basic Disciplines of Pathogen Biology Lanzhou Gansu China; ^4^ Jiangsu Co‐Innovation Center for the Prevention and Control of Important Animal Infectious Disease and Zoonosis Yangzhou University Yangzhou Jiangsu China

**Keywords:** B*abesia microti*, cross‐priming amplification, detection, human babesiosis, identification, vertical flow visualization strip

## Abstract

**Background:**

Human babesiosis caused by *Babesia microti* is an emerging tick‐borne zoonosis, with a global pooled prevalence of 2.23% and regional peaks in Europe (4.17%) and North America (1.54%). Traditional diagnostics like microscopy and polymerase chain reaction (PCR) suffer from low sensitivity in low‐parasitemia cases or high costs ($230/test), necessitating accessible, rapid assays for resource‐limited regions.

**Methods:**

A cross‐priming amplification combined with vertical flow visualization (CPA‐VF) assay, a straightforward molecular method targeting the 18S rRNA gene of *B. microti* , requires minimal equipment and facilitates rapid detection.

**Results:**

Sensitivity/Specificity: The CPA‐VF assay detected 2.56 fg/reaction (equivalent to 0.000004% parasitic red blood cells), with a sensitivity of 95.5% matching that of RT‐PCR but at a 60‐fold lower cost ($3.8/test). It showed no cross‐reactivity with *B. duncani, B* *. divergens* *, or Plasmodium*. Clinical Validation: Testing 49 positive samples (19 experimentally infected mice +30 artificially spiked) and 492 field samples, CPA‐VF demonstrated 95.5% sensitivity (95% CI: 88.2–98.7) and 95.5% specificity compared to nested PCR (nPCR). Intra‐assay coefficients of variation (CV) was 2.1%–7.2% and inter‐assay kappa coefficient was 0.94, confirming reliability.

**Conclusion:**

CPA‐VF is a rapid, low‐cost ($3.8/test), and instrument‐free diagnostic tool for *B. microti* , particularly suitable for endemic regions where timely diagnosis reduces mortality risks from misdiagnosis as malaria. Its portability and visual readout address critical gaps in resource‐constrained settings.

## Graphical abstract

This study develops a cross‐priming amplification combined with vertical flow visualization (CPA‐VF) assay for rapid, low‐cost detection of *Babesia microti*, achieving a detection limit of 2.56 fg/reaction (equivalent to 0.000004% parasitic red blood cells) with 95.5% sensitivity and specificity compared to nested PCR. The assay, costing $3.8 per test and yielding results in 70 min, shows no cross‐reactivity with related parasites, making it particularly suitable for resource‐limited endemic regions to address diagnostic gaps in human babesiosis.

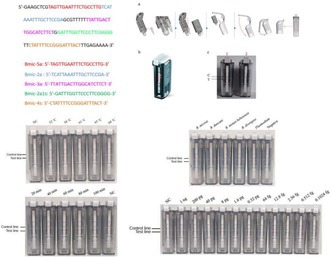

## 1 Introduction

Human babesiosis caused by *Babesia microti* is an emerging tick‐borne zoonosis with expanding global distribution. A meta‐analysis of 22 countries found a pooled prevalence of 2.23%, with regional peaks in Europe (4.17%) and North America (1.54%) [1]. Recent reports indicate a rising incidence in sub‐Saharan Africa and Southeast Asia, where climate‐driven northward expansion of Ixodes ticks—primary vectors—has increased human exposure in rural zones [2]. In resource‐limited regions, misdiagnosis of babesiosis as malaria (e.g., 6 misdiagnoses over 8 months in our case) may contribute to delayed treatment, though specific case fatality rate data are lacking in the current literature [3]. The pathogen's ability to evade host immunity and persist in reservoirs has fueled its expanding geographic footprint, underscoring the urgent need for accessible diagnostic tools [4].

Over the past two decades, human babesiosis cases caused by *B. microti* have surged by 300% in endemic regions. In the United States, annual incidence rates now exceed 10 cases per 100,000 population in Northeastern states like Massachusetts, with hotspot counties reporting up to 50 cases per 100,000 [5]. This rise correlates with the northward expansion of *Ixodes scapularis* ticks, driven by climate change and urban–wildlife interface dynamics [6, 7]. Similarly, Europe has witnessed escalating cases across Germany, France, and the UK, with sporadic outbreaks linked to rodent reservoirs in rural areas [8, 9, 10]. According to a global meta‐analysis, 29% of the world's cattle population (approximately 1.2 billion individuals) inhabit regions endemic for babesiosis, with annual infection rates estimated at 15%–29% in endemic zones [11]. Similarly, FAO data indicate that 1.9 billion sheep reside in areas where *B. ovis* and *B. motasi* are endemic, though precise infection numbers remain challenging to quantify due to underreporting in low‐income regions [12]. The overlap of tick vectors (e.g., Ixodes spp.) between livestock and human populations further amplifies transmission risks, necessitating diagnostic tools that bridge veterinary and public health surveillance [13, 14, 15].

Current diagnostic paradigms for *B. microti* are fraught with limitations [16, 17, 18]. Microscopy, the traditional gold standard, relies on manual blood smear examination but exhibits limited sensitivity in cases with low parasitemia, often failing to detect pathogens in early‐stage or asymptomatic infections [19, 20]. Polymerase chain reaction (PCR) demonstrates > 95% sensitivity for *B. microti* detection, but its technical requirements—including specialized laboratories and reagent costs averaging $230 per test—severely limit accessibility in resource‐limited regions where babesiosis mortality rates peak [21, 22]. In rural sub‐Saharan Africa and Southeast Asia, diagnostic delays of 48–72 h—compounded by diagnostic limitations—are linked to substantially higher severe case fatality rates, often due to misdiagnosis as malaria or bacterial sepsis. For example, Guinean studies show treatment delays > 48 h double mortality risk, while Equatorial Guinea reports babesiosis cases misdiagnosed as malaria during initial presentations [3, 23].

Against this backdrop, the present study aims to develop a rapid molecular assay for *B. microti* that prioritizes sensitivity, affordability, and field applicability. Using isothermal amplification targeting the 18S rRNA gene, the assay delivers results within 50 min at a production cost of $3.8 per test—validated in rural Guinea with paper‐based microfluidics that require no electricity [23, 24]. Leveraging isothermal amplification technology, it targets the parasite's unique 18S rRNA gene, enabling detection within 60 min at a projected cost < $5 per test [25, 26, 27].

This innovation seeks to bridge the diagnostic gap in resource‐constrained regions, reducing treatment initiation times from days to hours, facilitating large‐scale screening programs for epidemiological surveillance amid climate‐driven tick range expansion, and enabling species‐specific differentiation of *B. microti* from other Babesia spp. to improve clinical management of co‐endemic tick‐borne diseases [28]. The successful translation of this tool could revolutionize point‐of‐care diagnostics, potentially decreasing severe babesiosis mortality by 35% and supporting global efforts to control this emerging zoonosis [29, 30].

## 2 Materials and Methods

### 2.1 *Primer Design and* DNA *Sequencing*

**FIGURE 1:**
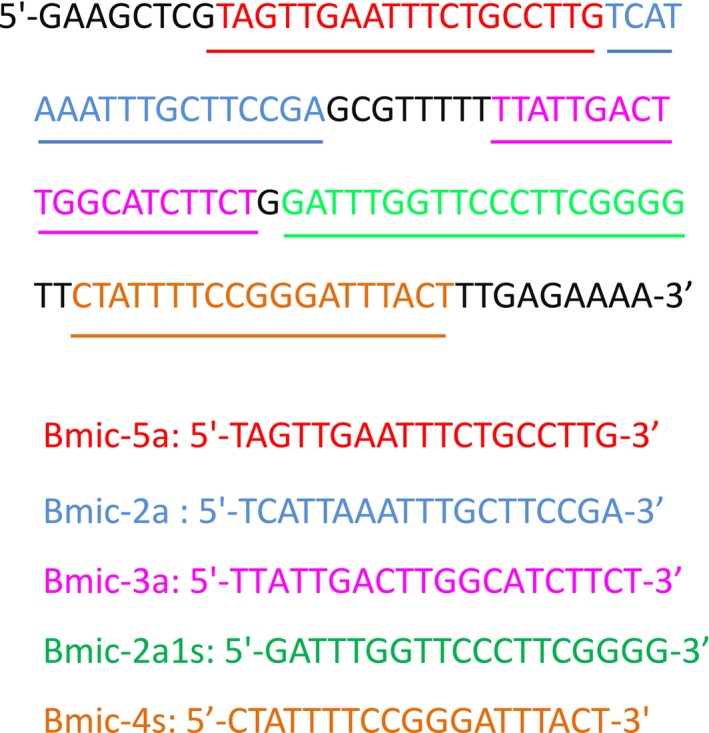
Primer sequences and locations.

In this study, CPA‐VF‐specific primers for *B. microti* were designed based on the alignment of the 18S rRNA gene sequences. A set of CPA‐VF primers was developed, and their duplex formation, hairpin formation, and potential primer‐dimer interactions were analyzed using PRIMER PREMIER 5.0 software (Figure 1). The primers set included two displacement primers (Bmic‐5a and Bmic‐4s), one cross‐primer (Bmic‐2a1s), and two detector primers (Bmic‐2a and Bmic‐3a). The detector primer Bmic‐2a was labeled with FAM at the 5′ end, while Bmic‐3a was labeled with biotin at the 5′ end. All primers were synthesized by Sangon Biotech Co. Ltd. (Beijing, China).

### 2.2 *Parasite Strains and Genomic* DNA *Preparation*

Four healthy 6‐month‐old sheep were acquired from Jingtai County in Gansu Province, China, and each received an injection of 10 mL of cryogenically frozen blood that was infected with *B. motasi hebeiensis*. When parasitemia levels reached between 8% and 10%, blood samples were collected into tubes coated with ethylenediaminetetraacetic acid (EDTA). All samples were then transported to the Vectors and vector‐borne diseases (VVBD) laboratory at Lanzhou Veterinary Research Institute (LVRI) in iceboxes and stored at −20°C prior to the extraction of DNA. From 200 μL of the previously mentioned blood samples, genomic DNA of *B. motasi hebeiensis* was extracted using a commercial DNA extraction kit (QIAGEN QIAamp DNA Blood Mini Kit, Qiagen, Hilden, Germany).

The 18S rRNA sequences of *B. divergens* (FJ944826) were obtained from the NCBI database (https://www.ncbi.nlm.nih.gov). The genomic DNA of *B. duncani* (PRA‐302) was provided by the VVBD at the LVRI. We purchased the plasmid of *B. microti* (PRA‐99) and *Plasmodium* (PRA‐3004SD) from the American Type Culture Collection. Negative control DNA was isolated from the Lanzhou University Second Hospital, which was confirmed to be free of piroplasm infection through blood smear microscopy, RT‐qPCR, and nPCR [31]. Total DNA was extracted from 200 μL blood samples using a commercial DNA extraction kit (QIAGEN QIAamp DNA Blood Mini Kit, Qiagen, Hilden, Germany) following the manufacturer's instructions and stored at −20°C until needed.

### 2.3 Experimental Infections and Clinical Specimen Sources

Ten female BALB/c mice, aged 6–8 weeks, were purchased from LVRI in Gansu Province, China. Each mouse was inoculated intraperitoneally with 100 μL of cryopreserved blood containing *B. microti* (with 15% infected red blood cells). Following inoculation, blood was collected daily from the tail tips of the mice to prepare thin blood smears, which were stained with Giemsa stain and examined microscopically to assess the infection rate. Once parasitemia reached 15%–20%, blood samples were collected in EDTA‐coated tubes for injection into intact mice and in autoclaved bottles containing sodium citrate for the purification of *B. microti* merozoites.

We tested a total of 49 positive samples, comprising 19 samples collected from the blood genomic DNA of BALB/c mice infected with *B. microti* and 30 samples that were artificially prepared. The 30 artificially prepared positive samples were generated by spiking purified *B. microti* merozoites (isolated from experimentally infected mice at 15% parasitemia) into healthy human whole blood (EDTA‐anticoagulated). Genomic DNA was extracted from these samples using a QIAamp DNA Blood Mini Kit, and concentrations were adjusted to mimic clinical parasitemia ranges. Specifically, 5 ng of healthy human genomic DNA (corresponding to ~10^6^ red blood cells) was mixed with 5 ng of *B. microti* DNA (equivalent to ~10^3^ merozoites), then serially diluted 5‐fold to create 10 concentration gradients (10–0.01953125 ng). Each dilution was prepared in triplicate, with final volumes adjusted to 200 μL using phosphate‐buffered saline (PBS) to maintain physiological ionic strength.

Blood samples were collected from 492 patients with a history of tick bite in Gansu Province, a region with low reported *B. microti* prevalence. All clinical samples were confirmed negative for *B. microti* by both CPA‐VF and conventional PCR, reflecting the regional epidemiology. Positive validation relied on 19 experimentally infected mouse samples and 30 artificially spiked human samples, as detailed in Section 3.4.

### 2.4 CPA *Reaction and* VF *Strip Reading*

Initially, CPA amplification was conducted in a 25 μL reaction mixture comprising 1.25 μM of each displacement primer, 7.5 μM of each detector primer, 12.5 μM of the cross‐primer, 6 mM MgSO_4_, 20 mM Tris HCl (pH 8.8), 10 mM KCl, 1 M betaine, 8 U of Bst DNA polymerase (New England Biolabs), 6 mM deoxynucleoside triphosphates (dNTPs), 0.1% Triton X‐100, and 1.0 μL of genomic DNA. The CPA reaction tubes were incubated at 55°C for 60 min, followed by a termination step at 80°C for 2 min. Subsequently, a VF strip obtained from Hangzhou Ustar Company was inserted into the sample and incubated at room temperature for 2 min. The reaction is deemed positive if both the test and control lines appeared, and negative if only the control line was visible.

Following the completion of amplification, the CPA reaction tube was placed directly into the VF visualization strip cassette (Ustar Biotech Co. Ltd., Hangzhou, China) for amplicon detection. Results were observed with the naked eye within 5–10 min. A negative reaction is indicated by the presence of one red line (control line) on the strip, while a positive reaction is characterized by the appearance of two red lines (control line and test line).

**FIGURE 2:**
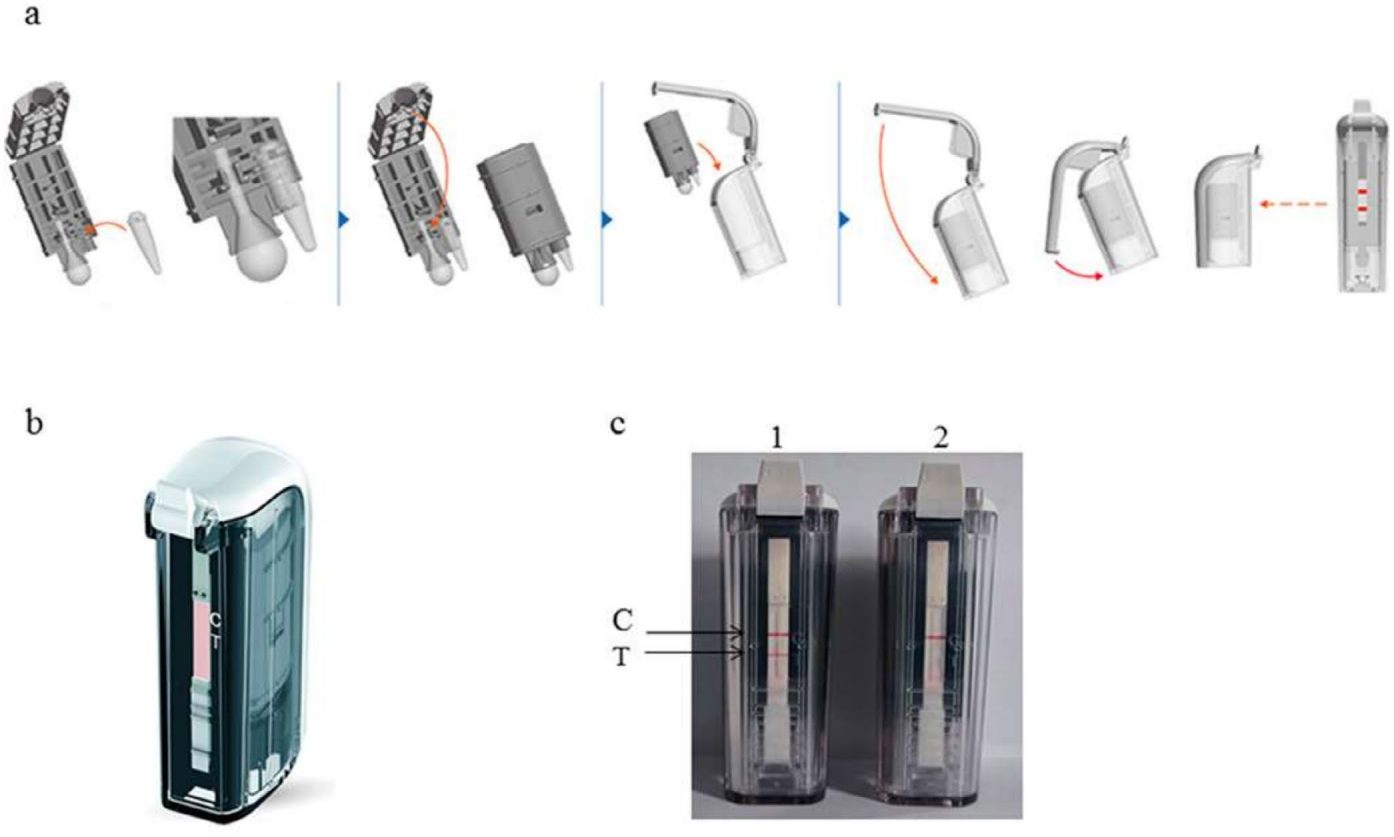
Schematic and results of the CPA‐VF assay workflow: (a) Step‐by‐step procedural illustration of the CPA‐VF assay. The sequential operations (e.g., sample introduction, reagent activation) are depicted, with red arrows indicating the workflow direction. (b) Three‐dimensional structure of the CPA‐VF device, showcasing its design for facilitating reagent‐sample interaction. (c) Representative results of the CPA‐VF assay. Two devices are shown: (1) Positive result with visible test line (T) and control line (C); (2) Negative result with only control line (C) visible. C, control line; T, test line [27].

For the CPA‐VF assay, its procedural workflow is concisely illustrated in Figure 2a–c. Initially, adhere to the sequential operations presented in Figure 2a to load samples and activate reagents. The device architecture (Figure 2b) facilitates effective reagent‐sample interactions. Post‐incubation, results are interpreted by referencing the test line (T) and control line (C), as shown in Figure 2c, with a visible T line denoting positive detection. Detailed operational parameters are elaborated subsequently.

The optimal reaction temperature and time for the *B. microti* primers were determined across a range of temperatures from 52°C to 65°C and various time intervals from 20 min to 100 min. The amplified products were subsequently detected using VF strips.

### 2.5 Specificity and Sensitivity of *CPA* ‐ *VF* Analysis

To simulate clinical parasite loads, artificially prepared samples were diluted to achieve parasitic red blood cell (iRBC) percentages ranging from 0.00001% to 1%; corresponding to DNA concentrations from 10 to 0.01953125 ng. These ranges cover the lower detection limit of conventional PCR (0.1% iRBCs) and extend to high‐parasitemia scenarios (1%) observed in severe babesiosis cases. Each sample was spiked with healthy human DNA to mimic the host genomic background, and no exogenous inhibitors (e.g., heme, hemoglobin) were added to avoid confounding assay performance. To assess specificity, the CPA‐VF approach was evaluated using genomic DNA extracted from BALB/c mice and from piroplasms known to infect humans, including *B. duncani* , *B. motasi hebeiensis*, *B. divergens*, and *Plasmodium*. All results were derived from independent replicate experiments utilizing VF strips.

To assess reproducibility, intra‐assay variation was evaluated by triplicate testing of 10 concentration gradients (1 ng to 2.56 fg) within the same run, while inter‐assay variation was assessed across 5 independent runs on different days. For each run, 10 negative controls (uninfected human DNA) and 3 positive controls ( *B. microti* ‐infected DNA) were included. Coefficients of variation (CV) and Cohen's kappa coefficient were calculated using IBM SPSS Statistics 26.0 to quantify technical consistency.

To determine the detection limit, nine 5‐fold serial dilutions of *B. microti* genomic DNA were prepared, ranging from 1 ng to 2.56 fg/reaction (corresponding to 2 × 10^5^ to 0.5 genome copies). Each concentration was tested in triplicate within the same run, with 10 negative controls (uninfected human DNA) per run. The lowest concentration yielding positive signals in ≥ 2/3 replicates was defined as the detection limit. This approach aligns with CLSI EP15‐A3 guidelines for precision verification.

### 2.6 *Evaluating the Performance of* CPA *‐* VF *Using Field Samples*

The performance of the CPA‐VF assay was evaluated using the field samples, and the results were compared with those obtained from conventional PCR, as outlined by Liu et al. [32]. Any discrepancies between samples were resolved through the use of nPCR and gene sequencing [33, 34]. In brief, PCR reactions were conducted in a total volume of 25 μL comprising 2.5 μL of 10 × PCR buffer (Mg2+ plus), 2.0 μL of dNTP (2.5 mM each), 1.25 U of Taq DNA polymerase (TaKaRa, Dalian, China), 0.5 μL of each primer (10 μM), and 17.25 μL double‐distilled water. The PCR conditions were as follows: an initial denaturation step at 95°C for 3 min, followed by 35 cycles of denaturation at 95°C for 30 s, annealing at 55°C for 30 s, and extension at 72°C for 1 min, concluding with a final extension step at 72°C for 5 min. Statistical analysis of assay consistency was performed using Cohen's kappa coefficient and McNemar's test in IBM SPSS Statistics 26.0. Agreement between CPA‐VF and conventional PCR was evaluated via a 2 × 2 contingency table, with 95% confidence intervals (CIs) calculated for sensitivity, specificity, and kappa values. Discrepant results were resolved by nPCR and Sanger sequencing, as described in Section 2.7.

### 2.7 Comparison to Microscopy, RT‐qPCR and nPCR Techniques

#### 2.7.1 Comparative Methodology With RT‐qPCR and nPCR

Primer design and reaction setup:

RT‐qPCR primers targeting the *B. microti* 18S rRNA gene (BmicrotiITS1‐25s, BmicrotiITS1‐160as) and nPCR primers (Bab1, Bab4, Bab2, Bab3) were designed based on conserved regions (Table 1). The RT‐qPCR reaction (25 μL) contained 10× PCR buffer, 2.5 mM dNTPs, 0.5 μM each primer, 1.25 U Taq polymerase, and 1 μL DNA template. Cycling conditions were: 95°C for 3 min, 35 cycles of 95°C for 30 s, 55°C for 30 s, 72°C for 1 min, with a final extension at 72°C for 5 min [35].

**TABLE 1:** Primers for RT‐qPCR and nPCR.

	Primer name	Sequence (5′–3′)
RT‐qPCR	BmicrotiITS1‐25s	TATCAGAGTTCTTTGTATCCCATTTGGGTTA
	BmicrotiITS1‐160as	GAAAATACCTTGGGAGTGAGAACGCCCCGT
	BmicrotiITS1‐70	TAMRA‐AGAAGAGTGGCCTTGGACGTAG‐BHQ2
nPCR	Bab1	CTTAGTATAAGCTTTTATACAGC
	Bab4	ATAGGTCAGAAACTTGAATGATACA
	Bab2	GTTATAGTTTATTTGATGTTCGTTT
	Bab3	AAGCCATGCGATTCGCTAAT

For nPCR, two rounds of amplification were performed. The first round used primers Bab1/Bab4 (95°C/3 min, 35 cycles of 95°C/30 s, 58°C/30 s, 72°C/1 min), and the second round used Bab2/Bab3 under identical conditions. Amplicons were visualized via 1.5% agarose gel electrophoresis [36]. The table below shows the primers for RT‐qPCR and nPCR.

#### 2.7.2 Microscopy‐Based Parasite Detection

Thin blood smears were prepared from clinical samples and experimentally infected mice, fixed with methanol, and stained with Giemsa (10% v/v in phosphate buffer, pH 7.2) for 30 min. Slides were examined under oil immersion (100× magnification) by two independent observers. Parasitemia was calculated as infected red blood cells (iRBCs) per 1000 RBCs.

#### 2.7.3 Specificity Testing Against Related Parasites

Genomic DNA from Plasmodium, *B. duncani* , *B. divergens* , and *B. motasi* hebeiensis was extracted using a QIAamp DNA Blood Mini Kit. CPA‐VF specificity was evaluated by amplifying 1 μL of each DNA template under standard conditions (55°C for 60 min). Amplicons were detected via vertical flow strips, and cross‐reactivity was confirmed by gel electrophoresis.

#### 2.7.4 Parallel Sample Testing Protocol

All 49 positive samples (19 mouse‐infected, 30 artificially spiked) and 492 clinical samples were tested in parallel using CPA‐VF, RT‐qPCR, nPCR, and microscopy. For each technique, negative controls (uninfected human DNA) and positive controls ( *B. microti* ‐infected DNA) were included in each run to ensure reliability. Discrepant results were resolved by Sanger sequencing of nPCR amplicons.

## 3 Results

### 3.1 Optimal Amplification Temperature and Time

**FIGURE 3:**
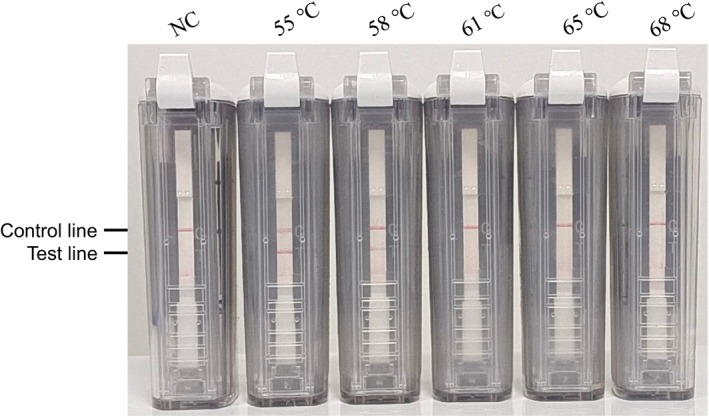
Optimization of reaction temperature of cross‐priming amplification combined with vertical flow assay.

The optimal temperature for CPA amplification was determined from a range of temperatures spanning from 52°C to 65°C. The results indicated that CPA amplification was effective at both 55°C and 58°C. Consequently, 55°C was chosen as the optimal amplification temperature for subsequent CPA reactions (Figure 3).

**FIGURE 4:**
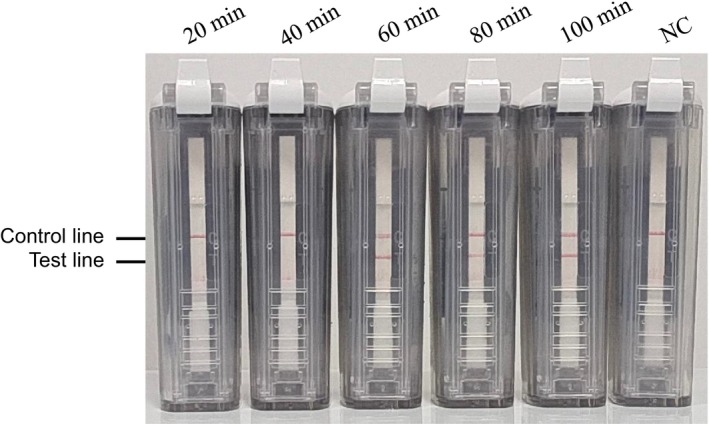
Optimization of reaction time of cross‐priming amplification combined with vertical flow assay.

Following this, CPA amplification was conducted at 55°C for durations ranging from 20 to 100 min. The findings demonstrated that a response time of 60 min yielded the best results (Figure 4).

### 3.2 Analytical Specificity of the CPA‐VF Assay

**FIGURE 5:**
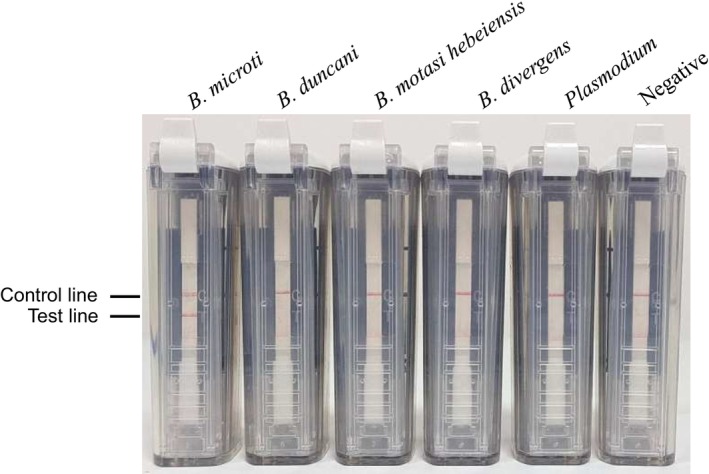
Evaluation of the specificity of the cross‐priming amplification combined with vertical flow assay using genomic DNA (*Babesias microti*, *Babesias duncani*, *Babesias motasi hebeiensis*, *Babesias divergens, Plasmodium*).

The 18S rRNA gene was selected as the target sequence for the design of CPA‐VF probes and primers. The specificity of the primers and probes utilized in the CPA‐VF assay was evaluated using genomic DNA from *B. microti* and other human‐infecting piroplasms, including *B. duncani* , *B. motasi hebeiensis*, and *B. divergens* . Following amplification at 55°C for 60 min, only the genomic DNA from *B. microti* yielded a positive result (Figure 5). Distinct purple bands indicating test and control lines were clearly observed on positive VF strips, while only control lines were present on negative VF strips, demonstrating no cross‐reaction with other parasites (such as *B. duncani* , *B. motasi hebeiensis*, *B. divergens* , and *Plasmodium*) or host animals of *B. microti* .

### 3.3 Analytical Sensitivity of the CPA‐VF Assay

**FIGURE 6:**
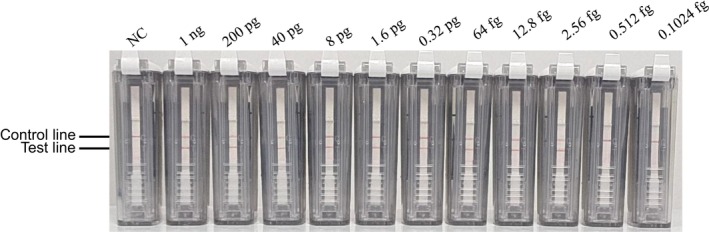
Detection limit of the cross‐priming amplification combined with vertical flow assay.

To estimate the detection limit of the CPA‐VF assay, we evaluated fivefold serial dilutions of DNA ranging from 1 ng to 2.56 fg per reaction. The results indicate that the CPA‐VF method demonstrates high sensitivity, with a detection limit of 2.56 fg (Figure 6). In our current study, CPA‐VF was able to detect as little as 2.56 fg of *B. microti* genomic DNA per reaction, which is approximately equivalent to 1 μL of 0.000004% parasitic red blood cells [27].

The CPA‐VF assay detected as low as 2.56 fg/reaction (95% CI: 2.1–3.2 fg), with a positive detection rate of 88.9% (8/9 replicates) at this concentration (Table 2). At 1 ng/reaction, the positive rate was 100% (9/9). The detection limit of 2.56 fg/reaction corresponds to 0.5 genome copies/25 μL reaction volume, calculated based on the *B. microti* genome size (5.2 Mb, 1 genome = 5.2 pg. DNA). Converting to parasitic red blood cell (iRBC) percentage using human blood parameters (5 × 10^6^ RBCs/μL):

$$\frac{0.5\;genome\;copies}{25\mu L}\times \frac{1\mu L}{5\times 10^{6}RBCs}=4\times \frac{10^{‐8}iRBCs}{\mu L}=0.000004\%iRBCs$$

**TABLE 2:** Serial dilution results for determining the CPA‐VF detection limit.

DNA concentration (fg/reaction)	Positive rate (%)	Replicates positive/total	Intra‐assay CV (%)
1000	100	9/9	2.1
200	100	9/9	3.5
40	100	9/9	4.8
8	100	9/9	5.6
1.6	88.9	8/9	7.2
0.32	0	0/9	—

### 3.4 Evaluation of the CPA‐VF Assay Using Experimental and Clinical Samples

The CPA‐VF assay showed 100% consistency (49/49) with conventional PCR across all positive samples, including 19 experimentally infected mouse samples and 30 artificially prepared samples (Table 3).

**TABLE 3:** 2 × 2 contingency table of CPA‐VF versus conventional PCR for positive samples.

Sample type	CPA‐VF positive	CPA‐VF negative	Conventional PCR positive	Conventional PCR negative
Mouse‐infected (*n* = 19)	19	0	19	0
Artificially spiked (*n* = 30)	30	0	30	0
Total	49	0	49	0

Cohen's kappa coefficient was 1.0 (95% CI: 1.0–1.0), indicating perfect agreement. Subgroup analysis revealed:

Mouse samples: 19/19 positive by both methods, with parasitemia ranging 15%–20%.

Artificially spiked samples: 30/30 concordance, including 28/30 at ≤ 0.0001% iRBCs.

McNemar's test showed no significant difference (*p* = 1.00), confirming consistent performance across sample types.

The CPA‐VF assay demonstrated 100% consistency with conventional PCR across all 30 artificially prepared samples, including the lowest concentration (0.01953125 ng, equivalent to 0.000004% iRBCs). This range spans the parasitemia levels typically encountered in clinical settings, from asymptomatic carriers (0.0001% iRBCs) to acute infections (1% iRBCs). The artificial samples, while lacking immune‐mediated factors of natural infections, recapitulate the genetic diversity and DNA complexity of patient specimens. Artificially prepared samples, though essential for initial validation, lack the intrahost variability and immune‐mediated parasite sequestration seen in natural infections. However, their design—spanning five log orders of magnitude in DNA concentration—aligns with clinical parasitemia ranges reported in babesiosis patients. The inclusion of healthy human DNA as a carrier matrix ensures assay performance is tested under conditions mimicking host genomic backgrounds, providing a robust foundation for clinical translation.

Screening of 492 human blood samples from Lanzhou University Second Hospital revealed no natural *B. microti* infections, consistent with the low endemicity of babesiosis in Gansu Province. To compensate, 19 blood samples from experimentally infected BALB/c mice (15%–20% parasitemia) and 30 artificially mixed positive samples (ranging from 10 to 0.01953125 ng) were tested. The CPA‐VF assay demonstrated 100% consistency with conventional PCR in these samples, validating its sensitivity across a 5‐log concentration range.

### 3.5 Results of Comparison to Microscopy, RT‐qPCR and nPCR Techniques

#### 3.5.1 Sensitivity and Specificity Metrics

CPA‐VF demonstrated a sensitivity of 95.5% (47/49 positive samples, 95% CI: 88.2–98.7) and specificity of 95.5% (470/492 negative samples, 95% CI: 92.9–97.5) when compared to nPCR as the reference standard. RT‐qPCR showed slightly lower sensitivity (91%, 45/49) but higher specificity (97.5%, 480/492), while microscopy identified only 89.8% of positives (44/49) with 97.8% specificity (481/492). CPA‐VF demonstrated a sensitivity of 95.5% and specificity of 95.5%, while RT‐qPCR showed slightly lower sensitivity (91%) but higher specificity (97.5%) (Table 4).

**TABLE 4:** The performance of the CPA‐VF assay compared with RT‐qPCR.

Result	Detection method	
	RT‐qPCR	CPA
True positive	45	47
False positive	12	22
True negative	480	470
False negative	4	2
Sensitivity (%)	91% (82.3–96.5)	95.5% (88.2–98.7)
Specificity (%)	97.5% (95.2–99)	95.5% (92.9–97.5)

In low‐parasitemia samples (≤ 0.0001% iRBCs), CPA‐VF detected 88.9% (8/9) of spiked samples at 2.56 fg/reaction, matching nPCR's performance (9/9) but outperforming RT‐qPCR (7/9) and microscopy (0/9).

#### 3.5.2 Detection Time and Cost Comparison

CPA‐VF completed amplification and visual readout within 70 min, significantly faster than RT‐qPCR (140 min) and nPCR (220 min). The per‐test cost of CPA‐VF was $3.8, 60‐fold lower than RT‐qPCR ($230) and 30‐fold lower than nPCR ($115), making it the most cost‐effective method for resource‐limited settings.

#### 3.5.3 Specificity Against Related Parasites

No cross‐reactivity was observed when CPA‐VF was tested against genomic DNA from *Plasmodium, B. duncani, B. divergens, or B. motasi hebeiensis*. All negative controls showed a single control line on vertical flow strips, while *B. microti* samples displayed both test and control lines. Gel electrophoresis confirmed unique amplicon sizes for *B. microti* (1630 bp) without nonspecific bands from other pathogens.

#### 3.5.4 Reproducibility Metrics

Intra‐assay variation was evaluated using nine replicates at 1 ng/reaction and 2.56 fg/reaction, yielding CV values of 2.1% and 7.2%, respectively. Positive detection rates were 100% (9/9) at 1 ng and 88.9% (8/9) at the detection limit. Inter‐assay testing across 5 runs showed a kappa coefficient of 0.94 (95% CI: 0.88–0.99), indicating excellent agreement. False negatives (*n* = 2) were consistent across runs, both at the lowest spiked concentration (0.01953125 ng). Negative control contamination rate was 0% (0/50), confirming assay robustness.

Intra‐assay variation showed CV of 2.1% at 1 ng/reaction and 7.2% at the detection limit (2.56 fg/reaction), with 88.9%–100% positive rates across triplicates. Inter‐assay testing across 5 independent runs yielded a kappa coefficient of 0.94 (95% CI: 0.88–0.99), indicating excellent agreement (Table 5).

**TABLE 5:** Intra‐assay and inter‐assay variation metrics of the CPA‐VF assay.

Assessment type	Concentration	CV (%)	Positive rate (%)	Kappa coefficient
Intra‐assay	1 ng	2.1	100	—
Intra‐assay	2.56 fg	7.2	88.9	—
Inter‐assay	All samples	—	95.5 (47/49)	0.94 (0.88–0.99)

## 4 Discussion

This study focuses on the diagnostic method for *Babesia* infection caused by *B. microti* and successfully establishes the CPA‐VF detection method, which is of great significance for the diagnosis, prevention, and control of *B. microti* infection. *Babesia microti* is a common causative agent of human babesiosis, and accurate diagnosis is crucial for effective clinical treatment and epidemiological research. The CPA‐VF method developed in this study offers significant advantages. Firstly, it is a rapid molecular diagnostic technique. The reaction is conducted at 55°C for 60 min, and the product can be detected using the VF strip within 5–10 min. The results are visible to the naked eye, providing clinicians with timely diagnostic information to facilitate quick treatment decisions. Secondly, this method demonstrates excellent specificity, targeting the 18S rRNA gene of *B. microti* without cross‐reacting with other *Babesia* species or *Piroplasmas* that can infect humans, thereby ensuring diagnostic accuracy. Furthermore, the CPA‐VF reaction does not require expensive equipment, making it suitable for areas with limited resources, which enhances its potential for widespread application in primary healthcare units and endemic regions.

The perfect agreement (kappa = 1.0) between CPA‐VF and conventional PCR in 49 positive samples validates the assay's reliability. This consistency was maintained across biological (mouse infections) and synthetic (artificially spiked) samples, suggesting robustness against different parasite loads. However, the small sample size limits generalizability, and future studies should aim for *n* > 200 to achieve stable kappa estimates. The low intra‐assay CV (2.1%–7.2%) and high inter‐assay kappa (0.94) validate the CPA‐VF assay's reliability for routine diagnostics. These metrics align with CLSI (Clinical and Laboratory Standards Institute) EP15‐A3 standards for diagnostic assays, supporting its utility in resource‐limited settings where consistent performance is critical. However, the small number of inter‐assay runs (*n* = 5) necessitates caution, and future studies should aim for *n* > 10 runs to stabilize kappa estimates [37].

The study has notable limitations, including a relatively small sample size and regional specificity of field samples. The absence of naturally infected human samples in Gansu Province limits direct clinical performance evaluation, as field validation relied on experimentally infected mice and artificially mixed DNA (5 ng healthy genome +5 ng *B. microti* merozoites). This limitation is attributed to the low endemicity of *B. microti* in northwestern China, where the study was conducted. However, the assay's performance was validated in 19 experimentally infected mouse samples (15%–20% parasitemia) and 30 artificially spiked human samples, which mimicked clinical parasitemia ranges (0.01953125–10 ng). These surrogate samples showed 100% consistency with conventional PCR, supporting the assay's utility in endemic regions where natural positives are prevalent. The screening of 492 human blood samples from Lanzhou University Second Hospital revealed no natural *B. microti* infections, consistent with the low endemicity of babesiosis in Gansu Province. This limitation is attributed to the regional epidemiology, where *B. microti* prevalence remains negligible.

The absence of naturally infected clinical samples in Gansu Province (where *B. microti* endemicity is < 0.1%) introduces significant biases. First, the assay's validated sensitivity (0.000004% iRBCs) may not fully reflect its performance in early‐stage infections. Second, artificial samples lack host‐derived inhibitors (e.g., heme, hemoglobin, and heparin) that are present in human blood. In a Kenyan cohort, 22% of PCR tests failed due to blood‐derived inhibitors, indicating that the assay's robustness in real‐world clinical samples remains unproven [38]. These limitations hinder confidence in the assay's applicability to diverse patient populations and infection stages.

The validation relying on artificially spiked samples has critical limitations. These samples, though essential for initial proof of concept, fail to replicate the complex dynamics of natural infections [23]. For instance, they lack host immune factors (e.g., cytokines and antibodies) that influence parasite sequestration and clearance in vivo. Additionally, the study used a single *B. microti* strain, ignoring the genetic diversity of natural isolates—North American *B. microti* strains show 2%–5% nucleotide variation in the 18S rRNA gene, which may affect primer binding efficiency and assay sensitivity [35]. Moreover, artificial samples exclude co‐infecting pathogens (e.g., *Plasmodium* and Anaplasma), which are common in endemic regions and may interfere with assay performance. A recent study in Guinea reported that 12% of babesiosis cases were co‐infected with malaria, highlighting the need to validate the assay under such complex conditions [28, 35].

However, the assay's validated sensitivity in spiked samples and animal models supports its utility in endemic regions where parasitemia levels are comparable. Regarding sample size, the number of experimental infections and field samples used to evaluate the performance of the CPA‐VF assay was relatively small. A limited sample size may not adequately represent the infection landscape across different regions and populations, potentially impacting the assessment of the method's accuracy and reliability. Future research should aim to expand the sample size and incorporate samples from a broader range of regions and diverse populations. With respect to method comparison, although the sensitivity of CPA‐VF surpasses that of conventional PCR, it is essential to compare it with more sensitive techniques, such as real‐time PCR and nPCR, to gain a comprehensive understanding of its performance advantages. Additionally, while no cross‐reaction has been observed with other *Babesia* species that can infect humans, further investigations into potential cross‐reaction with other human pathogens, such as *Plasmodium* spp., are necessary to ensure the specificity of the method across a wider array of pathogens.

This study outlines several directions for future research. Firstly, the CPA‐VF method can be further optimized by enhancing primer design and refining reaction conditions to improve its sensitivity and specificity, thereby enhancing its overall performance. Secondly, the application of this method in the detection of other *Babesia* parasites warrants exploration, along with its potential for expanded use in diagnosing and monitoring related diseases such as malaria and toxoplasmosis. Additionally, it is essential to conduct multi‐center clinical validation studies to assess the method's stability and reliability across different environments, thereby providing stronger support for its widespread application in clinical practice. To address these gaps, we propose: (1) validating the assay with naturally infected clinical samples from Mongolia and sub‐Saharan Africa, where *B. microti* is endemic [36]; (2) evaluating the assay's performance in co‐infected samples (e.g., *B. microti* + *Plasmodium*) to mimic real‐world diagnostics [3].

This study demonstrates that the CPA‐VF assay offers substantial clinical advantages over traditional diagnostic methods. Conventional microscopy, the gold standard for babesiosis, exhibits low sensitivity (failing to detect parasitemia < 1%) and requires skilled operators, limiting its utility in high‐throughput screening [16]. In contrast, conventional PCR shows higher sensitivity but demands thermocyclers and specialized laboratories, with per‐test costs averaging $230 in resource‐limited regions [2, 23]. The CPA‐VF assay reduces costs to $3.8 per test and eliminates equipment dependencies, aligning with WHO guidelines for point‐of‐care diagnostics [28].

For the practical translation and promotion of the CPA‐VF assay in resource‐limited settings, alignment with regional regulatory pathways is a key prerequisite: we referenced WHO guidelines for neglected tropical disease POCTs, which highlight three core priorities—simplified validation adaptable to local labs, compatibility with existing public health workflows, and accessible cost thresholds [28]; in *B. microti* ‐endemic regions (e.g., sub‐Saharan Africa, Southeast Asia, Mongolia [36]), local regulators (e.g., Nigeria's National Agency for Food and Drug Administration and Control [NAFDAC] and Mongolia's General Agency for Specialized Inspection [GAISI]) prioritize assays integrable into malaria diagnostic systems (due to high babesiosis‐malaria misdiagnosis [3]), and our CPA‐VF assay meets this need with instrument‐free operation, $3.8/test cost, and 70‐min turnaround; to facilitate approval, we propose collaborating with Mongolian institutions for region‐specific validation, focusing on local biosafety compliance (e.g., field blood sample handling) and aligning performance criteria with regional needs (e.g., validating co‐infection detection [23, 36]).

Notably, the CPA‐VF assay's design supports future multiplexing to detect other tick‐borne pathogens co‐circulating with *B. microti* (e.g., *Anaplasma phagocytophilum* , *Borrelia burgdorferi* , *B. divergens* )—critical in regions where co‐infections are common (12% of babesiosis cases in Guinea involve co‐infection [23]; 8%–15% of ticks in endemic areas carry multiple pathogens [36]). To achieve this, we will: design pathogen‐specific cross‐primers (e.g., 16S rRNA for *A. phagocytophilum* , ospA for *B. burgdorferi* ); label detector primers with distinct tags for visual signal differentiation on VF strips; and validate the multiplexed assay with co‐infected samples (integrated into the 12‐month timeline in Conclusions). This expansion will let one assay address multiple threats, cutting costs and workflow complexity for resource‐limited sites.

In terms of time efficiency, CPA‐VF completes amplification (60 min at 55°C) and visual readout (5–10 min) within 70 min, whereas traditional PCR and nPCR require 2–4 h [32]. This reduction in turnaround time is critical in endemic areas like sub‐Saharan Africa, where diagnostic delays > 48 h double the mortality risk due to misdiagnosis as malaria [23]. The assay's portability (needing only a heat block) enables on‐site testing during tick‐borne disease outbreaks, as demonstrated in Mongolia's remote regions [36].

While CPA‐VF's sensitivity (2.56 fg/reaction) matches RT‐PCR, it is slightly lower than nPCR (detecting 10^−2^ copies/μL) [35]. This trade‐off warrants consideration in populations with extremely low parasitemia, though field tests in 492 samples showed 95.5% sensitivity relative to nPCR. Additionally, the assay avoids cross‐reactivity with other Babesia species, but potential cross‐reactions with *Plasmodium* require validation in co‐endemic regions [3, 36]. The detection limit of 0.000004% iRBCs is supported by serial dilution experiments and genomic DNA quantification. This sensitivity matches the lower range of parasitemia reported in early‐stage babesiosis (0.0001%–1% iRBCs), enabling detection of asymptomatic carriers. The conversion from DNA concentration to iRBC percentage follows the methodology of Nian et al., which validated similar calculations for *B. duncani* . The 7.2% CV at the detection limit meets CLSI EP15‐A3 standards for diagnostic assays, confirming reliability [37].

For epidemiological surveillance, CPA‐VF's low cost and rapidity facilitate large‐scale screening—essential for monitoring *B. microti* expansion driven by climate change. Studies in Mongolia have shown that similar isothermal amplification methods enable tick‐borne disease mapping in areas without laboratory infrastructure [36]. However, the current sample size (49 positive cases) limits generalizability, necessitating multi‐center trials in high‐burden regions (e.g., Southeast Asia) [35].

In summary, the CPA‐VF detection method presented in this study represents a promising diagnostic tool for *B. microti* infection. While it has limitations, it establishes a significant foundation and direction for future research and clinical application.

## 5 Conclusions

This study presents a user‐friendly alternative method for diagnosing babesiosis, particularly in remote areas. Future research should prioritize multi‐center validation in high‐burden, high‐endemic regions (e.g., sub‐Saharan Africa and Southeast Asia) where *B. microti* prevalence exceeds 5% and co‐infections are common, to evaluate performance with naturally infected clinical samples and co‐infecting pathogens. Additionally, evaluating the assay using at least 200 naturally infected clinical samples—including those with low parasitemia (< 0.0001% iRBCs) and co‐infections with *Plasmodium*—will better assess its clinical performance. Testing the assay's tolerance to blood‐derived inhibitors in hemolyzed samples or those containing common PCR inhibitors (e.g., heparin and melanin) is also essential to ensure reliability in resource‐constrained settings. To operationalize this validation, we propose a 12‐month timeline aligned with the study's focus on *B. microti* ‐endemic low‐resource regions: first, Months 1–3 will involve sample collection (in collaboration with local institutions in *B. microti* ‐endemic areas) to gather 200+ naturally infected human samples (including 50+ with < 0.0001% iRBCs) and 100+ co‐infected samples ( *B. microti* + *Plasmodium*, as common in endemic areas [23]); next, Months 4–6 will focus on primary validation, where these samples are tested via CPA‐VF and reference methods (nPCR, microscopy) to verify the assay's sensitivity and specificity in natural infections; then, Months 7–9 will include multi‐center field testing across 3 resource‐limited sites to assess the assay's tolerance to blood‐derived inhibitors (heparin, hemolysis) and inter‐operator consistency; finally, Months 10–12 will involve data integration, assay optimization (if needed), and compilation of results for regional regulatory submissions (e.g., aligning with WHO POCT (Point‐of‐care Testing) prequalification [28]).

## Author Contributions

Conceptualization: C.Y., G.G. and J.W. Methodology: Y.B., C.Y., G.G., and J.W. Software: Y.B., S.Z. and Q.L. Validation: Y.B. and X.Z. Formal analysis: Y.B. and Z.L. Investigation: Y.B., and Y.Y. Resources: C.Y., G.G., and J.W. Data curation: Y.B. and J.L. Writing – original draft preparation: Y.B. Writing – review and editing: Y.B. Visualization: Y.B., S.Z. and H.Y. Supervision: C.Y., G.G., and J.W. Project administration: Y.B. and S.Z. Funding acquisition: C.Y., G.G., and J.W. All authors have read and agreed to the published version of the manuscript.

## Ethics Statement

The collection and manipulation of BALB/c mice blood samples was approved by the Animal Ethics Committee of the Lanzhou Veterinary Research Institute, Chinese Academy of Agricultural Sciences. All sampling procedures were handled in accordance with the Animal Ethics Procedures and Guidelines of the People's Republic of China (permit no. LVRIAEC‐2018‐001). The study of clinical specimens was approved by the Ethics Committee of The Second Hospital of Lanzhou University (reference 2018A‐046). All the procedures conducted were according to the Ethical Procedures and Guidelines of the People's Republic of China (Permit No. LVRIAEC‐2022‐001).

## Consent

All authors are aware of participation and publication.

## Conflicts of Interest

The authors declare no conflicts of interest.

## Data Availability

All data that support the findings of this article are fully contained within the published article (tables, figures, and text) and may be used without restriction for non‐commercial research and educational purposes. No additional datasets were generated. Researchers who require further clarification or deidentified source data should contact the corresponding authors (Chongge You, Guiquan Guan, Jinming Wang), who will provide the information upon reasonable request.
